# Cholesterol Depletion Alters Cardiomyocyte Subcellular Signaling and Increases Contractility

**DOI:** 10.1371/journal.pone.0154151

**Published:** 2016-07-21

**Authors:** Mohammed Z. Haque, Victoria J. McIntosh, Abdul B. Abou Samra, Ramzi M. Mohammad, Robert D. Lasley

**Affiliations:** 1 Interim Translational Research Institute, Department of Internal Medicine, Academic Health System, Hamad Medical Corporation, Doha, Qatar; 2 Hypertension and Vascular Research, Department of Internal Medicine, Henry Ford Hospital, 2799 West Grand Blvd., Detroit, MI 48202, United States of America; 3 Department of Physiology, Wayne State University School of Medicine, 1104 Elliman Bldg., 421 East Canfield, Detroit, MI 48201, United States of America; Institute of Biochemistry and Biotechnology, TAIWAN

## Abstract

Membrane cholesterol levels play an important factor in regulating cell function. Sarcolemmal cholesterol is concentrated in lipid rafts and caveolae, which are flask-shaped invaginations of the plasma membrane. The scaffolding protein caveolin permits the enrichment of cholesterol in caveolae, and caveolin interactions with numerous proteins regulate their function. The purpose of this study was to determine whether acute reductions in cardiomyocyte cholesterol levels alter subcellular protein kinase activation, intracellular Ca^2+^ and contractility. **Methods**: Ventricular myocytes, isolated from adult Sprague Dawley rats, were treated with the cholesterol reducing agent methyl-β-cyclodextrin (MβCD, 5 mM, 1 hr, room temperature). Total cellular cholesterol levels, caveolin-3 localization, subcellular, ERK and p38 mitogen activated protein kinase (MAPK) signaling, contractility, and [Ca^2+^]i were assessed. **Results**: Treatment with MβCD reduced cholesterol levels by ~45 and shifted caveolin-3 from cytoskeleton and triton-insoluble fractions to the triton-soluble fraction, and increased ERK isoform phosphorylation in cytoskeletal, cytosolic, triton-soluble and triton-insoluble membrane fractions without altering their subcellular distributions. In contrast the primary effect of MβCD was on p38 subcellular distribution of p38α with little effect on p38 phosphorylation. Cholesterol depletion increased cardiomyocyte twitch amplitude and the rates of shortening and relaxation in conjunction with increased diastolic and systolic [Ca^2+^]i. **Conclusions**: These results indicate that acute reductions in membrane cholesterol levels differentially modulate basal cardiomyocyte subcellular MAPK signaling, as well as increasing [Ca^2+^]_i_ and contractility.

## Introduction

Cholesterol is a key lipid component of cell and organelle membranes that regulates membrane fluidity. Cholesterol is not evenly distributed throughout cell membranes but is concentrated with sphingolipids in lipid rafts. [1] Caveolae, specialized forms of lipid rafts that contain the scaffolding protein caveolin, are flask-shaped invaginations in the plasma membrane yielding membrane microdomains that serve to compartmentalize signal transduction. [2],[3],[4] In addition to being enriched in cholesterol lipid rafts and caveolae, are characterized by their resistance to detergent (Triton X-100) solubilization. [5],[6],[7] Caveolin, which has a high affinity for cholesterol, binds to numerous proteins in various cell types. [2],[3],[4] Evidence that the function of these proteins is directly modulated by their localization in caveolae or association with caveolin is based on the results of an increasing number of studies utilizing cholesterol depletion and/or caveolin knockout. The reduction of membrane free cholesterol levels, with agents such as methyl-β-cyclodextrin (MβCD), disrupts the structure of lipid rafts and caveolae leading to altered cell signaling and function. [8–13] MβCD is a water-soluble, cyclic heptasaccharide with a hydrophobic cavity that has been shown to reduce plasma membrane cholesterol levels, while having little effect on the extraction of phospholipids. [14],[15]

It has been reported that MβCD decreases adult cardiomyocyte cholesterol levels, [16],[17] and reduces ischemic tolerance and blocks opioid receptor-mediated protection of ischemic cardiomyocytes and isolated perfused hearts. [18],[19] However the effects of reductions in cholesterol on adult cardiomyocyte signaling have not been reported. There is also evidence that numerous proteins in cardiac myocytes, which regulate calcium handling, are localized in lipid rafts and/or co-localize with caveolin-3. [20],[21],[22],[23] Reports nearly 25 years ago indicated that in vitro function of various cardiac ion pumps, such as the sarcolemmal Na^+^-Ca^2+^ exchanger and the Na^+^-K^+^ ATPase, could be modulated by changes in cholesterol levels, [24,25] and cholesterol depletion alters L-type calcium current in cardiomyocytes. [16],[17],[25] Despite these reports there have been very few studies examining the effects of cholesterol reduction on basal cardiomyocyte contractility. The purpose of this study was therefore to determine the effects of acute cholesterol depletion with methyl-β-cyclodextrin (MβCD) on cardiomyocyte subcellular signaling and function.

## Materials and Methods

All animals in this study received humane care according to guidelines in “The Principles of Laboratory Animal Care” formulated by The National Society for Medical Research and the National Institutes of Health “Guide for the Care and Use of Laboratory Animals” (National Institutes of Health Publication No. 86–23, Revised 1996). Animals were also used in accordance with the guidelines of the Wayne State University Animal Care and Use Committee.

### Isolation of adult rat ventricular myocytes

Ventricular myocytes were enzymatically dissociated from adult male Sprague-Dawley rats (280–320 g). Rats were heparinized (500 U ip) and deeply anesthetized with pentobarbital sodium (65 mg/kg ip). The hearts were then rapidly excised and retrogradely perfused as described previously.[26] Cardiomyocytes were suspended in experimental buffer containing 130 mM NaCl, 4.7 mM KCl, 1.2 mM KH_2_PO4, 1.2 mM MgSO4, 1 mM CaCl_2_, 10 mM HEPES, 11.0 mM glucose, creatinine 5 mM, taurine 5 mM, pH 7.4. This protocol yields > 75% viable, rod-shaped cardiomyocytes. After extracellular calcium was restored to 1 mM, the myocytes were allowed to sit at room temperature for 1 hour prior to initiating experimental protocols.

#### Experimental treatments

After the 1 hour recovery period cardiomyocytes were gravity settled and then resuspended in fresh HEPES-buffered medium (1 mM calcium, pH 7.4), medium supplemented with 5 mM methyl-β-cyclodextrin (MβCD), or medium containing 5 mM MβCD complexed with cholesterol 125 μg/ml. The MβCD-cholesterol complex was made by initially dissolving cholesterol (Sigma Aldrich) in a 1:1 solution of chloroform and methanol. Seventy-five μl of this solution was dried down and then reconstituted in medium supplemented with warmed 5 mM MβCD. This solution was sonicated for 1 hour and then kept at 37°C overnight prior to use. After the 1 hour treatments myocytes were gravity settled and the MβCD or MβCD-cholesterol containing medium was removed. Cell yields that were used for signaling studies were immediately processed. Myocytes used for contractility studies were resuspended in fresh HEPES-buffered medium.

#### Cholesterol measurements

Separate groups of myocytes (3–4 per group) were used to measure total cholesterol contents. Whole cell lysates were generated by incubating the homogenized myocytes with 1% triton for 30 minutes on ice. This suspension was then centrifuged at 10,000 g for 10 minutes and both triton-soluble and triton insoluble fractions were collected and stored at -80°C until analysis. Total cholesterol levels were analyzed with a commercially available kit (Wako Chemicals, Richmond, VA). Cholesterol values were normalized to protein concentration in each sample.

#### Cardiomyocyte subcellular fractionation

After the 1 hour treatments myocytes (n = 4–5 isolations/group) were gravity settled for 5 minutes, the buffer was removed and the cells were quickly resuspended in ice-cold homogenization buffer (320 mM sucrose, 10 mM KCl, 1.5 mM MgSO4, 1 mM EDTA, 1 mM EGTA, 20 mM HEPES, pH 7.4, 1 mM dithiothreitol, 0.1 mg/ml PMSF, 45 μg/μl aprotinin, 0.5 mM beta-glycerophosphate, 1 mM sodium vanadate). All of the following steps were conducted on ice or at 4°C. The cells were homogenized with a dounce homogenizer (15–20 strokes) and sonicated three times in 5 sec bursts, and the resulting homogenate was centrifuged for 10 minutes at 750 g. The resulting pellet was then mixed well with ~ 800 μl homogenization buffer containing 1% triton X-100, incubated on ice for 30 minutes, and then centrifuged at 10,000 g for 10 minutes. The supernatant was collected and denoted as the cytoskeletal (CySk) fraction. The supernatant obtained from the 750 g spin was centrifuged at 100,000 g for 30 minutes, and the resulting supernatant was saved as the cytosolic (Cyto) fraction. The pellet from the 100,000 g spin was resuspended in homogenization buffer containing 1% triton X-100 and incubated on ice for 30 minutes. This sample was then centrifuged again for 30 minutes at 100,000 g. The final supernatant represents the triton-soluble membrane fraction (Sol). The triton-insoluble pellet was dissolved in homogenization buffer (Insol). Total protein in each fraction was determined with a BCA protein assay reagent kit (Thermo Scientific, Rockford, IL).

### Western blot analysis

Protein separation by SDS-PAGE and western blot analysis were performed using methods that we have previously described. [27] Protein samples (10–40 μg) were separated and transferred to nitrocellulose membranes (Bio-Rad, Hercules, CA). Ponceau S staining was used to verify equal protein loads. For the kinase studies membranes were probed with polyclonal antibodies recognizing dually phosphorylated forms of p38 (from Santa Cruz Biotechnology, Santa Cruz, CA), ERK1/2). Membranes were then stripped and blotted with polyclonal antibodies for p38α and ERK. Additional blots (10 μg protein) were probed with monoclonal antibodies for caveolin-3 (BD Pharmingen) and cytochrome-*c* oxidase (Invitrogen) or a polyclonal antibody for α-actinin (Santa Cruz). Bound antibodies were visualized by enhanced chemiluminescence (Amersham, Piscataway, NJ). Immunoreactive bands were quantified with UN-SCAN-IT gel digitizing software (Silk Scientific, Inc., Orem, UT).

### Cardiomyocyte contractility and calcium studies

Small aliquots of cardiomyocytes (n = 20–30/group) were loaded in the dark with Fluo-4-acetoxymethyl ester (Fluo 4, 1 μM, room temperature, 20 min) in the presence of probenecid (0.5 mM). The myocytes were washed with HEPES medium to remove extracellular dye, and incubated in the dark for an additional 20 minutes to allow for intracellular deacetylation. An aliquot of dye-loaded myocytes was then allowed to settle on laminin-coated coverslips and placed in a temperature-controlled recording chamber (RC-24 chamber, TC-324B temperature controller, Warner Instrument, Hamden, CT) on the stage of an IX-70 Olympus inverted microscope (Olympus America, Melville, NY). Cells were suffused with normal HEPES buffer (pH 7.4 at 37°C) at a flow rate of 1 ml/min, field stimulated (0.5 Hz, SD9 stimulator, Grass Instruments, Quincy, MA) and imaged by CCD camera.

Myocyte twitch strength and kinetics were monitored and analyzed using sarcomere length detection (SARACQ-SarcLen^™^) and fluorescence software (IonWizard) obtained from IonOptix Corp. (Milton, MA). A region of 15–20 sarcomeres within the myocytes were identified and monitored to measure contractility, the rate of cell-shortening, and the rate of relaxation as described by Wang et al. [28] Myocytes were allowed to reach steady-state twitching values for 5 minutes before recording contractility data. Fluo-4 fluorescence values were recorded in the same cells. An excitation wavelength of 490 nm was used, and emission fluorescence was collected at 520 ± 5 nm. To reduce excitation-dependent oxidation of the dye, a neutral density filter (ND12) was placed in the path of the excitation source (75W xenon arc lamp). All cell fluorescence values were background corrected and expressed as Fluo-4 fluorescence intensities.

### Statistical Analysis

Results are expressed as means ± SE. Effects of MβCD treatment were compared to control (untreated) group. Western blot results were analyzed using the Student’s t-test. Myocyte twitch and intracellular calcium results were analyzed with one-way ANOVA followed by Tukey’s post-hoc test. A P value < 0.05 was considered statistically significant. All data described in this manuscript are available in the Supplemental information files.

## Results

### Cholesterol results

Treatment with 5 mM MβCD for 1 hour had no effect on cardiomyocyte viability or morphology. Treatment with MβCD decreased total cellular cholesterol concentration from a control value of 9.0 ± 1.4 to 4.6 ± 0.3 (μg cholesterol/mg protein; p < 0.05). Exposure of additional myocyte suspensions to MβCD + cholesterol for 1 hour increased total cholesterol levels to 21.0 ± 2.2 (μg cholesterol/mg protein).

### Characterization of subcellular fractions

**Fig 1:**
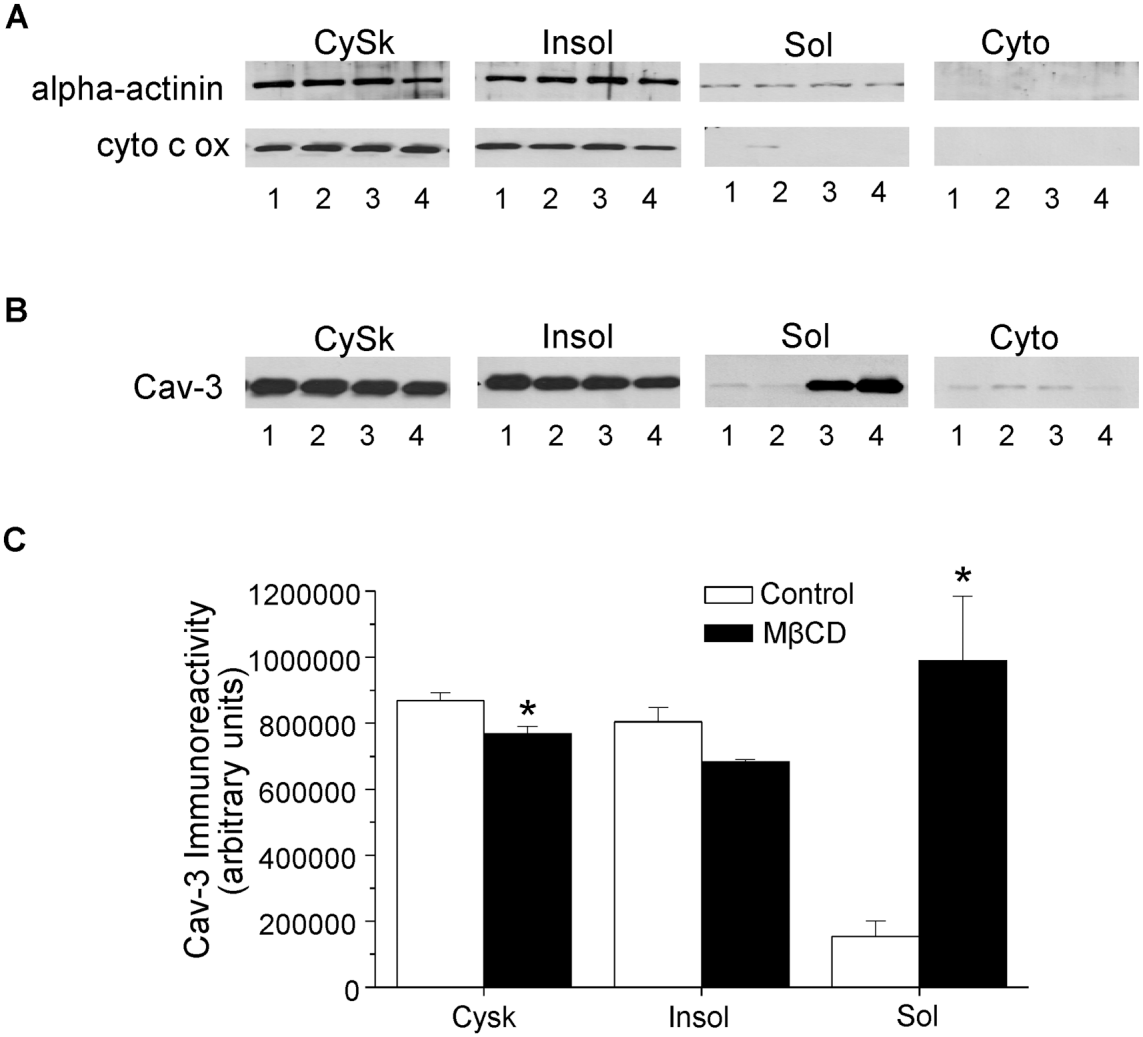
Characterization of subcellular fractions in control and MβCD treated myocytes. (1A) A representative blot demonstrating the subcellular distribution of α-actinin and cytochrome c oxidase subunit IV (cyt c ox) in time control myocytes (lanes 1 and 2) and myocytes treated with MβCD (lanes 3 and 4). (1B) Western blot demonstrating the effect of MβCD (lanes 3–4) on the subcellular distribution of caveolin-3 (cav-3); time control myocytes are in lanes 1–2. (1C) Summarized results showing the distribution of cav-3 in control and MβCD treated myocytes. Cardiomyocytes were treated with the cholesterol-depleting agent MβCD (5 mM) for 1 hour at room temperature. Subcellular fractions are: cytoskeletal (CySk), triton-insoluble (Insol), triton-soluble (Sol) and cytosol (Cyto). In the cytosol fraction caveolin-3 was not different and negligible compared to other fractions and therefore not given in the bar graph. *p < 0.05 vs. time control, n = 3–4/group.

The characterization of the four subcellular fractions is shown in the representative blots of Fig 1A. Alpha-actinin, a sarcomeric protein enriched in the Z lines, exhibited significant expression in the pellet generated by the low-speed spin (designated as cytoskeletal (CySk)) and the triton-insoluble membrane fraction, with much less in the triton-soluble membranes. The marker for the inner mitochondrial membrane, cytochrome c oxidase subunit IV, was present in similar amounts in the triton-insoluble and cytoskeletal fractions, but not in the triton-soluble fraction. Consistent with reports that caveolin-3 is resistant to triton solubilization, [2],[3],[4],[5],[6],[7] the majority of membrane caveolin-3 was expressed in the triton-insoluble membranes (84%) compared to triton-soluble membranes. MβCD treatment had no effect on the subcellular distribution of alpha-actinin or cytochrome c oxidase subunit IV, but did alter the subcellular localization of caveolin-3 (Fig 1B and 1C), which decreased in the cytoskeletal and triton-insoluble membrane fractions, but increased in the triton-soluble membrane fraction.

### Effect of cholesterol reduction on ERK MAPK

**Fig 2:**
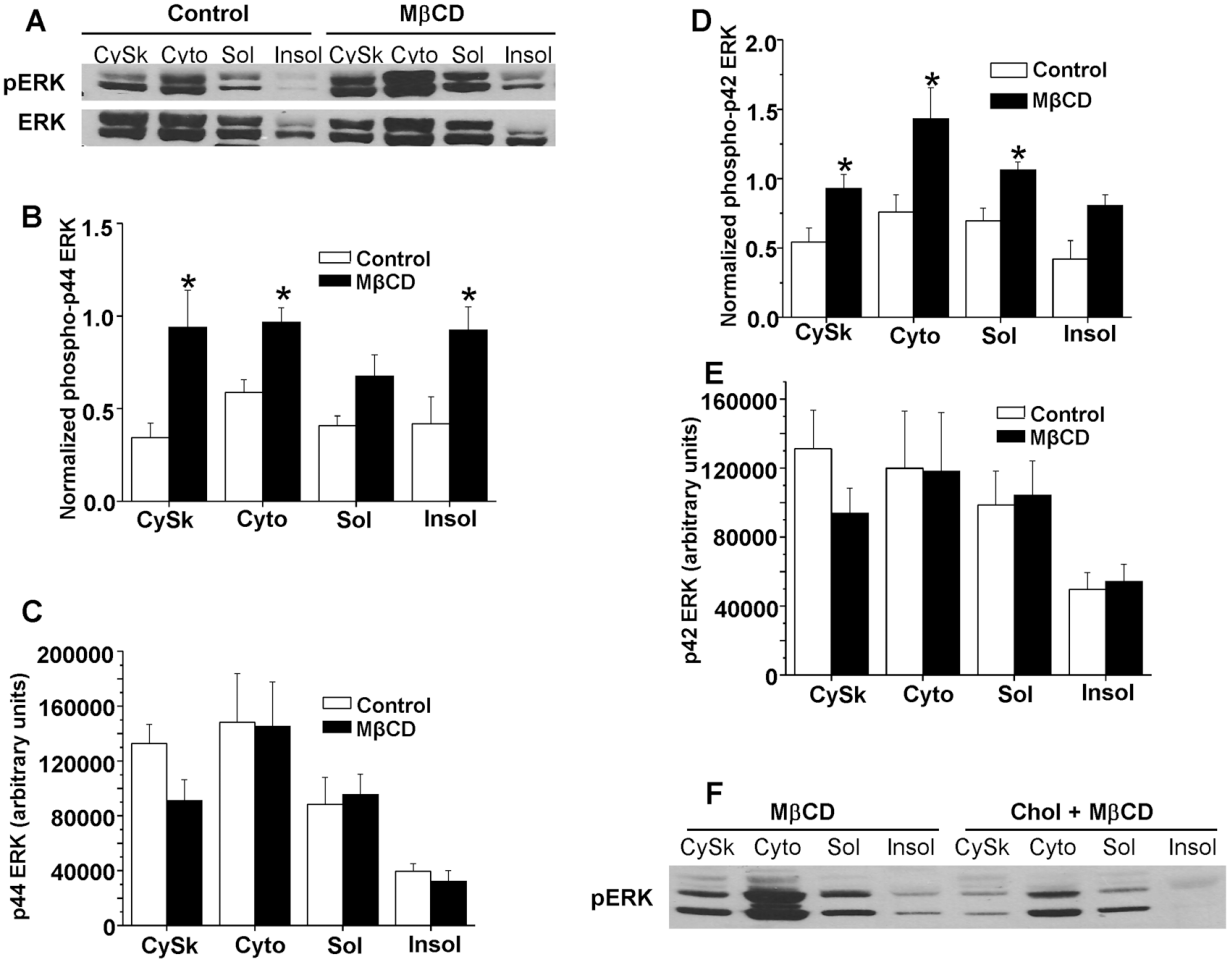
Subcellular analysis of the effect of MβCD on p44/p42 ERK in rat cardiomyocytes. (2A) A representative blot of dually-phosphorylated and total p44 and p42 ERK in control and MβCD treated cells. (2B) The effects of MβCD on the subcellular distribution of phosphorylated and (2C) total p44 ERK. Phosphorylated p44 values shown in (2B) are normalized to total p44 ERK in each fraction. Normalized phosphorylated and total p42 ERK are shown in Figs (2D) and (2E), respectively. *p < 0.05 vs. time control, n = 5/group. Fig (2F) is a representative western blot showing the effect of enhancing cardiomyocyte cholesterol with MβCD + cholesterol on ERK phosphorylation.

The effects of the cholesterol reducing agent, MβCD, on cardiomyocyte subcellular ERK are shown in Fig 2A–2F. Fig 2A is a representative blot of subcellular ERK isoform phosphorylation and total expression in time control and MβCD treated cardiomyocytes. Fig 2B shows the summarized results for p44 phosphorylation normalized to total p44 expression in each fraction. Treatment with MβCD significantly increased phosphorylation of p44 ERK in the cytoskeletal (2.7 fold), cytosolic (1.7 fold) and triton-insoluble fractions (2.4 fold) compared to time control myocytes. P44 ERK phosphorylation appeared to be elevated in the triton-soluble membrane fraction, but this effect was not statistically significant (p = 0.07). Although there were differences in the subcellular localization of the 44 kD ERK isoform, MβCD treatment had no statistically significant effect on its redistribution (Fig 2C). We found ~10% of cardiomyocyte p44 ERK was present in triton-insoluble membranes, whereas 70% of this isoform was expressed in the cytoskeletal and cytosolic fractions.

Similar results were observed with respect to MβCD’s effects on p42 ERK. Cholesterol reduction increased p42 ERK phosphorylation in all four subcellular fractions, although the increase in the triton-insoluble fraction was not statistically significant. Cardiomyocytes treated with MβCD exhibited 1.7, 1.9 and 1.5 fold increases in p42 ERK phosphorylation (normalized to p42 expression) in cytoskeletal, cytosolic and triton-soluble membrane fractions, respectively, compared to control myocytes (Fig 2D). These effects were independent of changes in isoform distribution (Fig 2E), although p42 ERK distribution did show differences in subcellular localization. As shown in the representative blot in Fig 2F the acute increase in myocyte cholesterol levels blocked the effects of cholesterol depletion on phospho-ERK.

### Effect of MβCD on subcellular p38 MAPK

**Fig 3:**
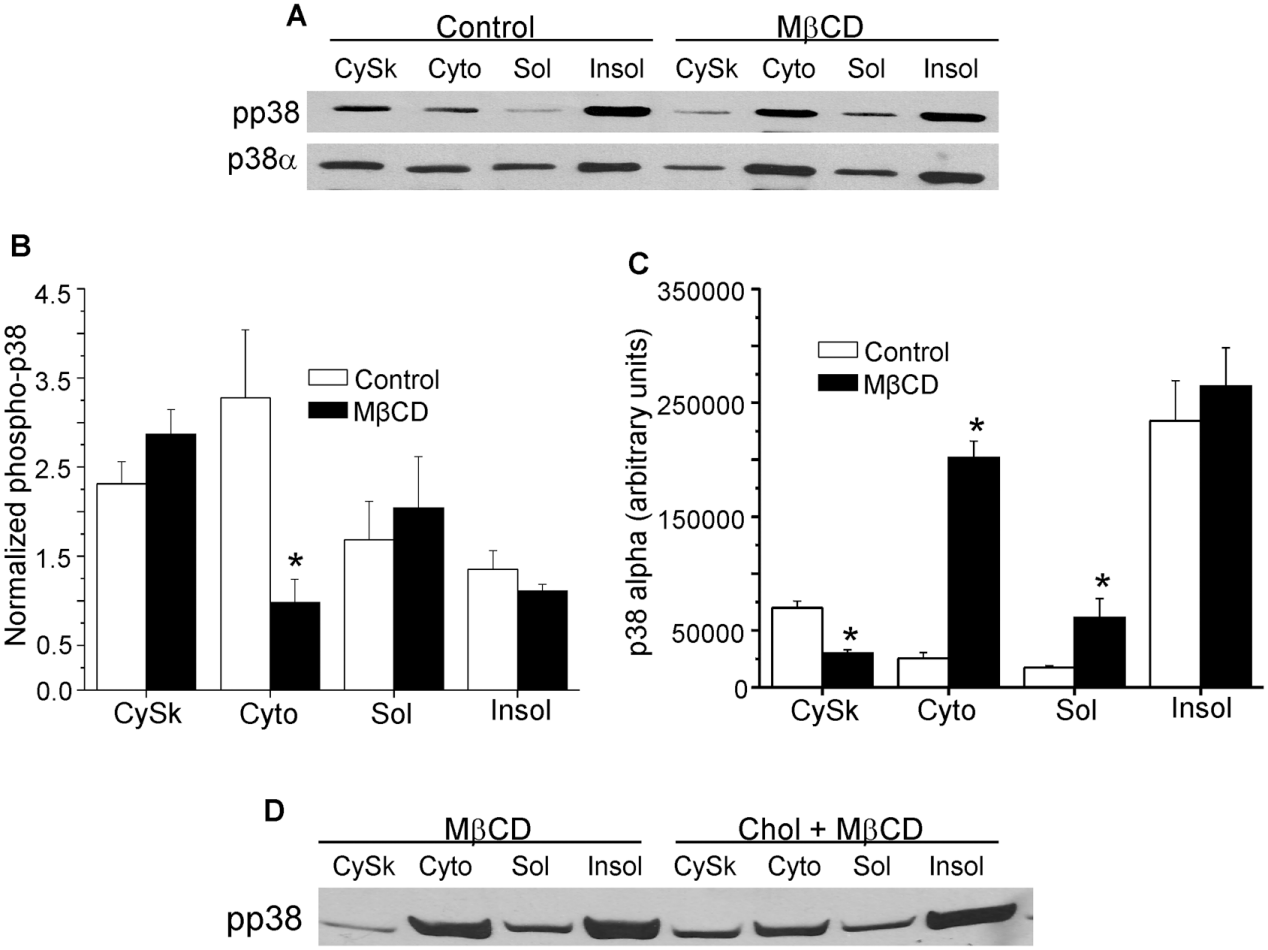
Effect of cholesterol depletion with MβCD on subcellular p38 MAPK. (3A) Representative western blot showing expression of dually-phosphorylated and total p38α MAPK in cytoskeletal (Cytsk), cytosolic (Cyto), triton-soluble (Sol) and triton-insoluble (Insol) fractions. Summarized blot results for dually phosphorylated p38 normalized to total p38α and total p38α are shown in Figs (3B) and (3C), respectively. *p < 0.05 vs. control, n = 4/group. Fig (3D) shows a pp38 western blot in myocytes treated with MβCD + cholesterol.

Fig 3 illustrates the effects of the cholesterol reducing agent on p38 MAPK. Representative blots of phospho-p38 and total p38α are shown in Fig 3A, and summarized results are shown in Fig 3B and 3C, respectively. Treatment of cardiomyocytes with MβCD induced ~ a 45% decrease in p38 phosphorylation normalized to p38α in the cytosolic fraction (Fig 3B). Under normal conditions p38α MAPK is expressed to the greatest extent in triton-insoluble membranes, with significantly less distribution in cytoskeletal, cytosolic and triton-soluble fractions (Fig 3C). Treatment with MβCD decreased p38α expression in the low-speed pellet (cytoskeletal fraction) and increased p38α in the cytosolic and triton-soluble fractions (both effects p < 0.05, Fig 3C). There was no effect of the cholesterol reducing agent on p38α levels in triton-insoluble membranes. As shown in the blot in Fig 3D the acute increase in myocyte cholesterol levels blocked the effects of cholesterol depletion on p38 phosphorylation.

### Effects of changes in cardiomyocyte cholesterol on contractility and intracellular calcium

**Fig 4:**
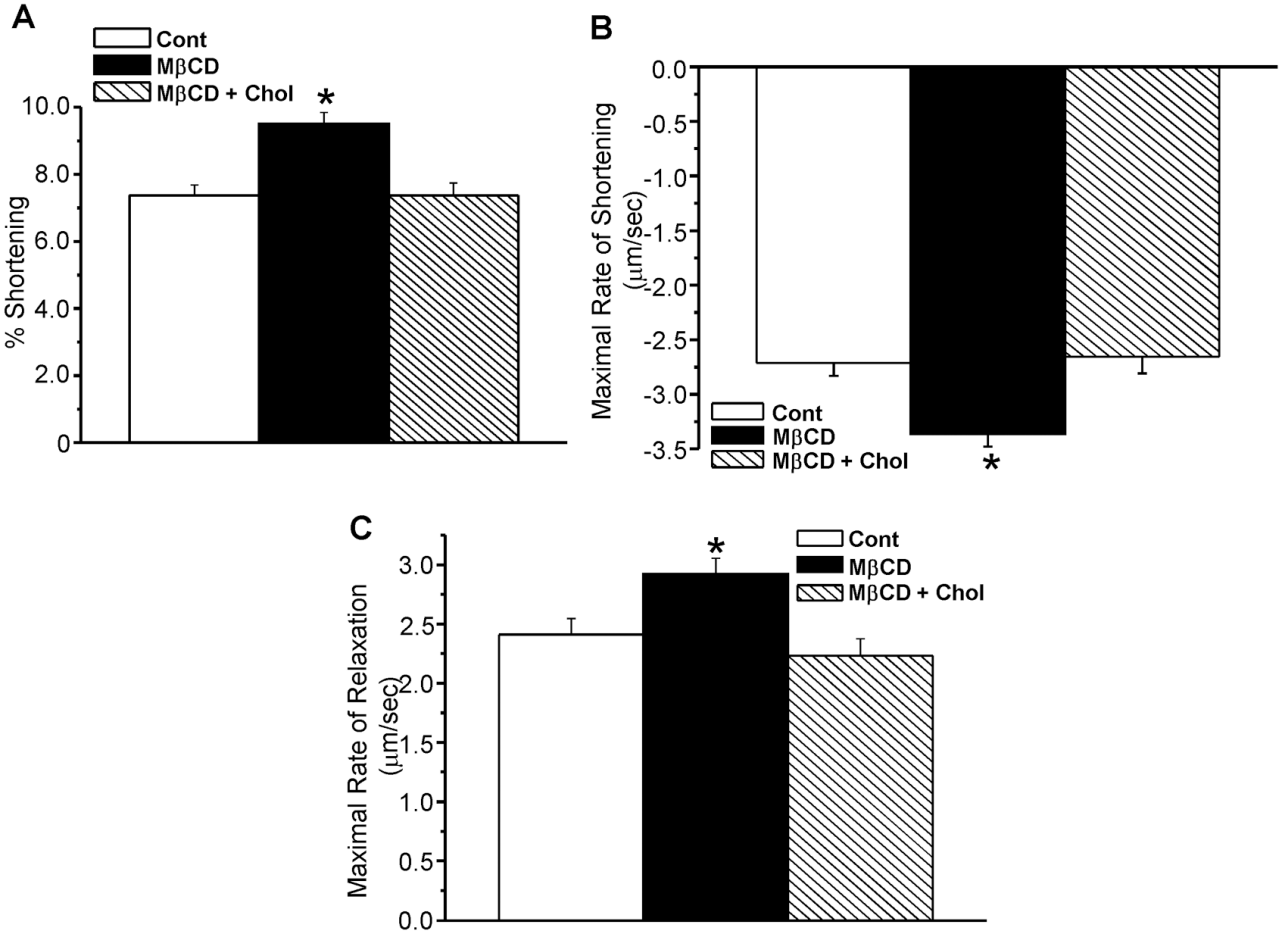
Effects of cholesterol depletion with MβCD and cholesterol enrichment with MβCD + cholesterol on cardiomyocyte contractility. (4A) Contractility of percentage shortening, (4B) maximal rate of shortening, and (4C) maximal rate of relaxation. Contractility parameters were calculated as described in the text. * p<0.05 vs. control, n = 20–30/group.

The effects of MβCD and cholesterol + MβCD on (A) cell shortening, (B) maximal rate of shortening and (C) maximal rate of relaxation are shown in Fig 4 and S4 Table. Fig 4A indicates that treatment with MβCD increased myocyte contractility by 30% compared to time-control myocytes. Cardiomyocytes treated with MβCD + cholesterol exhibited cell shortening values similar to control cardiomyocytes. The rate of shortening (Fig 4B) was increased by 24% with MβCD, whereas this parameter was unaffected by MβCD + cholesterol treatment. Similarly cholesterol depletion increased the rate of myocyte re-lengthening (Fig 4C), whereas cholesterol loading had no effect on this parameter.

**Fig 5:**
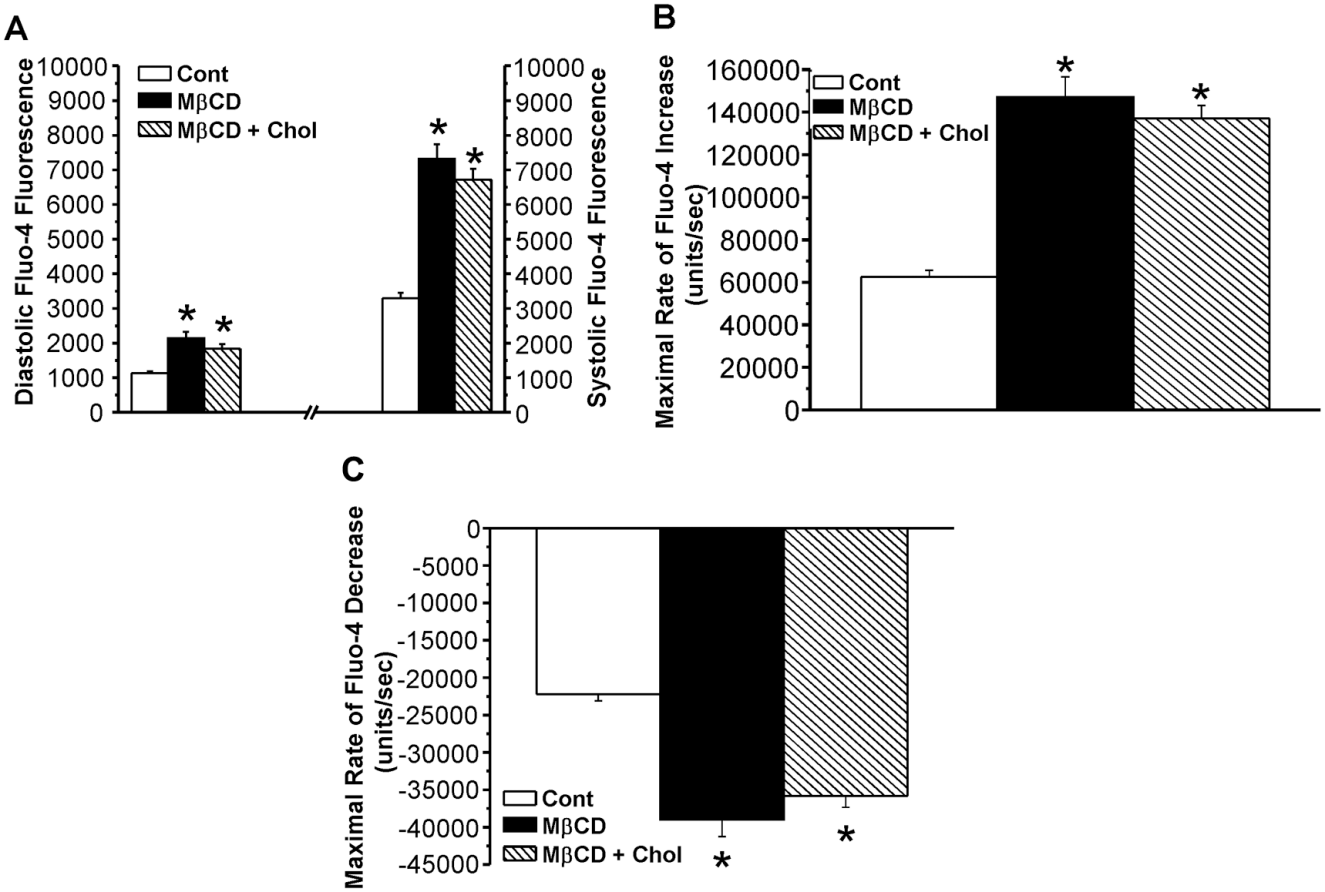
Effects of MβCD and MβCD + cholesterol on intracellular calcium. (5A) Diastolic and systolic Fluo-4 fluorescence values. (5B) Maximal rate of Fluo-4 increase during systole, and (5C) maximal rate of Fluo-4 decrease during diastole. * p<0.05 vs. control, n = 20–30/group.

Fig 5 and S5 Table summarize the effects of MβCD and cholesterol + MβCD on Fluo-4 fluorescence as an index of cardiomyocyte free intracellular calcium. In contrast to the effects on contractility both treatments exerted nearly identical effects on diastolic and systolic Fluo-4 values (Fig 5A), the maximal rate of rise of the Ca transient (Fig 5B), and the maximal rate of decline of the transient (Fig 5C). Treatment with MβCD increased diastolic Fluo-4 levels 90% and systolic Fluo-4 121% compared to time-controls; treatment with MβCD + cholesterol increased these parameters 60% and 103%, respectively (Fig 5A). Both treatments increased the maximal rate of rise of the Ca transient by 120% and the maximal rate of decline of the transient by ~ 70% compared to control values (Fig 5B).

## Discussion

The results of the present study indicate that acute cholesterol depletion alters subcellular cardiomyocyte MAPK signaling, and increases intracellular calcium and contractility. Treatment with the cholesterol-reducing agent MβCD shifted caveolin-3 from cytoskeleton and triton-insoluble fractions to the triton-soluble fraction, and increased subcellular ERK phosphorylation independent of the subcellular localization of this MAPK. In contrast the primary effect of cholesterol depletion on p38 MAPK was a redistribution of this protein. Cholesterol depletion increased cardiomyocyte contractility and the kinetics of shortening and relaxation in association with increases in the amplitude and kinetics of the calcium transient. Treatment with a MβCD + cholesterol complex increased cardiomyocyte cholesterol levels, which appeared to block the MβCD effects on signaling and contractility. In contrast cholesterol loading exerted similar effects as cholesterol depletion on intracellular calcium. These results indicate that cardiomyocyte signaling and excitation-contraction coupling are very sensitive to acute changes in membrane cholesterol levels.

Plasma membrane cholesterol is enriched in microdomains referred to as lipid rafts and caveolae. Caveolae contain the scaffolding protein caveolin, and caveolin-3 is the primary caveolin isoform expressed in adult cardiomyocytes. The high affinity of caveolin for cholesterol helps maintain the structure of caveolae, and caveolin binds to numerous proteins regulating their function, [2]–[4],[29] and changes in cholesterol or caveolin levels have been shown to alter the function of numerous proteins in several cell types. [8]-[11],[30] Although it has been reported that cholesterol depletion blocks opioid receptor-mediated cardioprotection, [18],[19] the mechanism for this effect has not been determined.

Our findings indicate that treatment with MβCD decreased total cardiomyocyte cholesterol levels by ~45% and altered the subcellular distribution of caveolin-3. These effects were associated with an increase in the phosphorylation of both ERK isoforms in multiple subcellular fractions without any changes in the subcellular distribution of either isoform. Our findings that alterations in caveolin-3 subcellular localization are associated with increased ERK phosphorylation are consistent with reports that ERK possesses a caveolin-3 binding domain, [31] and caveolin-3 deficient mice exhibit increased myocardial ERK phosphorylation. [30] Furthermore Wang et al reported a high molecular weight complex between ERK and two protein phosphatases, the tyrosine phosphatase HePTM and the serine/threonine PP2a, is cholesterol sensitive. [32] Disruption of this complex by cholesterol depletion in human fibroblasts and HeLa cells led to activation of ERK. This same mechanism may occur in cardiomyocytes, since it has been reported that PP2a co-immunoprecipitates with ERK in these cells. [33] In addition we previously reported significant PP2A activity in cardiomyocyte insoluble membrane fractions. [34]

In contrast to the effects on ERK, cholesterol depletion had little effect on p38 phosphorylation, but rather acted primarily by altering subcellular distribution of p38α. The levels of this kinase decreased in the cytoskeletal fraction, but increased in the cytosolic and triton-soluble membrane fractions. Normalization of the subcellular phospho-p38 levels to total p38α revealed that these changes in p38α distribution resulted in decreased p38 phosphorylation in the cytosolic fraction, but no changes in the three other subcellular fractions. These observations are similar to those of Zeidan et al [35], who reported that MβCD (5 mM, 1 hr, 37°C) had no effect on p38 phosphorylation (ERK phosphorylation was increased) in neonatal rat cardiomyocyte cell lysates. Cholesterol depletion had no effect in the triton-insoluble membranes. This result was somewhat surprising given our recent observation that > 80% of p38α in the triton-insoluble fraction could be immunoprecipitated with caveolin-3 IgG (3). However this fraction exhibited the greatest p38 phosphorylation under basal conditions, and p38α levels in this fraction were not altered. Our findings suggest that cholesterol homeostasis differentially regulates p38 and ERK MAPKs, and that these effects may be dependent on the subcellular localization of these kinases.

Additional observations from the present study indicate that cholesterol depletion in cardiomyocytes is also associated with altered excitation-contraction coupling. Cardiomyocyte contractility was increased by cholesterol depletion in association with increases in diastolic and systolic [Ca^2+^]i as measured by Fluo-4 fluorescence. The maximal rates of contraction and relaxation, as well as the rates of increase and decrease of the [Ca^2+^]i transients, were also increased following MβCD treatment. Calaghan and White [17] reported that cholesterol depletion with MβCD decreased both contractility and [Ca^2+^]i in adult rat cardiomyocytes, results opposite to what we observed. We used the same concentration of MβCD as these authors, although we limited our exposure time to 1 hour, whereas they used 2 hours exposure. Their longer duration of MβCD exposure could have induced a greater reduction in cholesterol than we observed; since they did not measure cholesterol levels, as we did, this explanation cannot be discounted. The only other apparent difference between the two studies is that we conducted our contractility and calcium imaging studies at 37°C, whereas Calaghan and White [17] used room temperature conditions. Although this difference may seem trivial, there are multiple reports of the temperature dependence of cardiomyocyte contractility and calcium handling, and in some cases opposite effects are seen at room temperature vs. 37°C. [36],[37],[38],[39]

Our observations that cholesterol depletion altered cardiomyocyte intracellular calcium concentration are supported by several previous reports. It has been reported that L-type calcium channels and a population of ryanodine receptors are present in caveolae and/or co-localize with caveolin-3. [16],[20],[22] Tsujikawa et at [16] reported that, when MβCD (10 mM, 10 min, room temperature) was delivered to isolated rabbit cardiomyocytes by patch pipette, cholesterol levels decreased by 50% and inward calcium current density at positive potentials significantly increased, suggesting that cholesterol depletion modulates L-type calcium channels. Agarwal et al [40] reported increased L-type calcium channel activity following MβCD treatment in adult rat ventricular myocytes, an effect that could partially explain the increased systolic [Ca^2+^]i that we observed in the present study. These same authors observed that MβCD treated myocytes exhibited increased contractile responses to the β agonist, isoproterenol, although they did not report the effects of MβCD on basal contractility. These findings by Agarwal et al [40] conflict with those of Calaghan and White [17], who reported that MβCD treatment did not alter isoproterenol effects on myocyte contractility or [Ca^2+^]i. The results of additional studies indicate that several proteins regulating diastolic [Ca^2+^]i, such as the sarcolemmal Ca^2+^-ATPase and the Na^+^-Ca^2+^ exchanger, are present in caveolae. [21],[41] Prior studies indicated that the activities of the Na^+^-Ca^2+^-exchanger and the Na^+^-K^+^-ATPase in canine cardiac sarcolemmal vesicles were dependent on cholesterol levels, whereas the SR Ca^2+^-ATPase was not altered by changes in cholesterol. [24],[25] These effects could explain the increases in systolic and diastolic [Ca^2+^]i that we observed following MβCD treatment.

Treatment with MβCD + cholesterol doubled cardiomyocyte cholesterol levels, an effect which resulted in restoration of contractility and twitch kinetics to control levels. These effects on contractility occurred despite increased diastolic and systolic [Ca^2+^]i and kinetics of the transient. Although these findings were unexpected it must be pointed out that although MβCD has been shown to preferentially deplete sarcolemmal cholesterol, [14],[15] it is likely that the MβCD + cholesterol complex does not act as selectively. Cholesterol enrichment has been reported to increase Na^+^-Ca^2+^ exchange activity but decrease sarcolemmal Ca^2+^-Mg^2+^ ATPase activity in sarcolemmal vesicles. [24] Cholesterol depletion and enrichment both decrease sarcolemmal Na^+^-K^+^ ATPase activity. [24] Thus acute cholesterol enrichment appears to exert differential effects on [Ca^2+^]i and cardiomyocyte contractility.

Our present findings that subcellular protein kinase signaling in ventricular myocytes can be regulated by acute changes in cholesterol level appear to have direct relevance to myocardial ischemia. More than 35 years ago Rouslin et al [42] reported that regional myocardial ischemia in pigs was associated with a significant increase in mitochondrial cholesterol content. Venter et al [43] subsequently reported that myocardial ischemia in isolated rat hearts was associated with a decrease in sarcolemmal cholesterol and an increase in mitochondrial cholesterol. We also reported that myocardial ischemia/reperfusion-induced activation of p38 and ERK MAPKs was associated with changes in subcellular cholesterol levels and caveolin-3 distribution. [27] These previous reports, in combination with our present observations with MβCD, suggest that reductions and/or the redistribution of intracellular cholesterol may play an important role in cardiomyocyte subcellular protein kinase signaling and excitation-contraction coupling. These data suggest that acute reductions and increases in cholesterol exert significant effects on basal signaling and contractility, effects which must be taken into account when interpreting the effects of cholesterol modulation on cardiomyocyte function.

## Supporting Information

S1 Table Data for graphs in Fig 1C. (XLSX)

S2 Table Data for graphs in Fig 2B, 2C, 2D and 2E. (XLSX)

S3 Table Data for graphs in Fig 3B and 3C. (XLSX)

S4 Table Data for graphs in Fig 4A, 4B and 4C. (XLSX)

S5 Table Data for graphs in Fig 5A, 5B and 5C. (XLSX)
